# PD-1/PD-L1 Inhibitor-Associated Myocarditis: Epidemiology, Characteristics, Diagnosis, Treatment, and Potential Mechanism

**DOI:** 10.3389/fphar.2022.835510

**Published:** 2022-04-19

**Authors:** Hao Dong, Yihang Qi, Xiangyi Kong, Zhongzhao Wang, Yi Fang, Jing Wang

**Affiliations:** Department of Breast Surgical Oncology, National Cancer Center/National Clinical Research Center for Cancer/Cancer Hospital, Chinese Academy of Medical Sciences and Peking Union Medical College, Beijing, 100021, China

**Keywords:** immunotherapy, myocarditis, epidemiology, mechanism, PD-1/PD-L1 inhibitors

## Abstract

Immune checkpoint inhibitors (ICIs) induce T-cell activation against cancer cells, and due to their anti-tumor function in multiple cancers, ICIs have been considered an important option for oncotherapy. PD-1/PD-L1 inhibitors are now widely used as ICIs for many types of cancers in clinical practices. Myocarditis induced by anti-PD-1/PD-L1 agents is uncommon but shows potentially fatal toxicity. In this review, we attempted to conclude the incidence, characteristics, diagnosis, and treatments, as well as illustrate the potential pathogenesis from the perspectives of T-lymphocyte infiltration, disturbance of regulatory T cells, cytokines, macrophage-mediated inflammatory response, and synergistic effect of PD-1/PD-L1 and CTLA4.

## 1 Introduction

**FIGURE 1:**
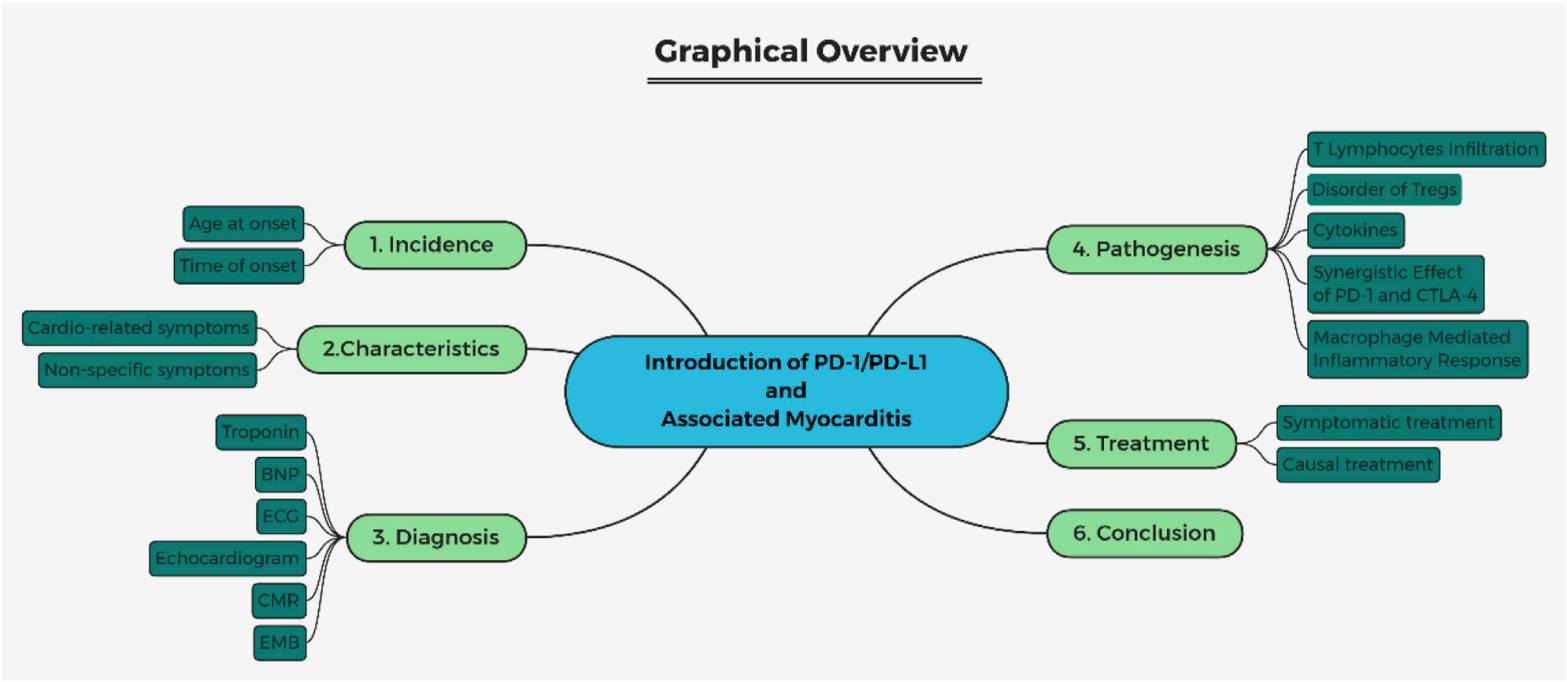
Graphical structure overview. BNP, brain natriuretic peptide; ECG, electrocardiogram; CMR, cardiovascular magnetic resonance; EMB, endomyocardial biopsy.

PD-1/PD-L1, the immune checkpoints, produce a negative regulation of T-cell function that maintains the balance between T-cell activation, tolerance, and immune-mediated tissue damage and also control the induction and maintenance of immune tolerance in the tumor microenvironment (Li et al., 2018; Han et al., 2020). PD-1, also called CD279 and homologous to the CD28, is a 288-amino acid type I transmembrane protein receptor (95 amino acids composing intracellular domain), which is encoded by the PDCD 1 gene that is located on chromosome 2. PD-L1, also known as CD 274, is a 290-amino acid protein receptor (with cytoplasmic 30 residues), which is encoded by the CD274 gene that is located on chromosome 9 (Ishida et al., 1992; Freeman et al., 2000; Keir et al., 2008). PD-1 is expressed on so many functional cells, like macrophages, activated T lymphocytes, natural killer lymphocytes, dendritic cells (DCs), B lymphocytes, and monocytes, whereas the ligand of PD-1 is usually located on activated B cells and T cells, DCs, and some epithelial cells. In the tumor microenvironment, PD-1 can be highly expressed on tumor-specific T cells, and PD-L1 can also be expressed on tumor cells (Ahmadzadeh et al., 2009). The combination of PD-1/PD-L1 can lead to attenuation of activated tumor-specific T cells, causing adaptive immunity and maintaining immune tolerance in the tumor microenvironment (Bivona et al., 2003; Patsoukis et al., 2013; Wang and Wu, 2020). Tumor cells can utilize this immune homeostasis to escape the immune surveillance, promoting malignant tumor dilation (Li et al., 2018; Salmaninejad et al., 2018). With the understanding of the immune-related mechanism in the process of tumor genesis and development, immunotherapy has become an important part of comprehensive tumor therapy. Recently, the immune checkpoint inhibitors have been widely used in tumor immunotherapy. Monoclonal antibody treatments of programmed cell death-1 (PD-1) and programmed death-ligand 1 (PD-L1) have shown promising therapeutic effects in many cancers, including melanoma, non-small cell lung cancer (NSCLC), renal cell carcinoma, head and neck squamous cell carcinoma, urothelial cancer, refractory Hodgkin lymphoma, breast cancer, and malignancies with microsatellite instability (Motzer et al., 2015; Ferris et al., 2016; Bellmunt et al., 2017; Lee et al., 2017; Hu J. R. et al., 2019). However, T cells may simultaneously cause damage to normal tissues, which subsequently result in immune-related adverse events (irAEs) (Cousin and Italiano, 2016; Hu J. R. et al., 2019; Su et al., 2020). As PD-1/PD-L1 inhibitors induce the immune cells to attack normal tissues, many organs can be damaged, such as skin, lungs, heart, muscles, bowels, liver, endocrine tissues, eyes, kidneys, as well as central nervous system (Naidoo et al., 2015; Ferris et al., 2016; Bellmunt et al., 2017; Hahn et al., 2017; Qin et al., 2019; Su et al., 2020). According to meta-analyses, myocarditis is one of the less common but with more fatal types in immune checkpoint inhibitor treatment. About 1% of patients were reported with myocarditis, but deaths due to immune myocarditis accounted for a large proportion of deaths induced by irAEs, ranging from 27 to 46% (Wang et al., 2018; Rubio-Infante et al., 2021). Myocarditis refers to the alterations in the number and function of lymphocyte subsets and macrophages and antibody-mediated injury in cardiomyocytes (Sagar et al., 2012). Also, T lymphocyte infiltration can be found in most cases with PD-1/PD-L1 inhibitor-associated myocarditis. Like normal myocarditis, it can also manifest diverse symptoms, like cardio-specific or non-specific symptoms (Sagar et al., 2012; Martín et al., 2021; Thompson et al., 2021). But the incidence, diagnosis, and other detailed information of it have been still unclear (Norwood et al., 2017). There is a need to better understand the clinical characteristics of myocarditis with high lethality, particularly, in an environment where more and more cancers can be treated by PD-1/PD-L1 inhibitors. In this article, we aimed to review the incidence, pathogenesis, clinical characteristics, diagnosis, and treatment of cardiotoxicity of PD-1/PD-L1 inhibitors. PD-1/PD-L1 inhibitor-associated myocarditis is a kind of immune checkpoint inhibitor-related myocarditis. Under the circumstances that there is no study specialized for PD-1/PD-L1-related myocarditis, this review has summarized the current cohort studies, case reports, or case series that focus on PD-1/PD-L1 inhibitors or have a large proportion of PD-1/PD-L1 inhibitors in immune checkpoint inhibitor treatment, with the proportion ranging from 63 to 86% (Escudier et al., 2017; Mahmood et al., 2018; Salem et al., 2018; Wang et al., 2018; Awadalla et al., 2020). The structure of this review is shown in Figure 1.

## 2 Incidence

Nestor et al. conducted an immune checkpoint inhibitor pharmacovigilance study and reported a number of 4,401 cardiac disorder records (4.2% of the total 104,276 records concerning adverse drug reactions) based on VigiBase (the WHO’s pharmacovigilance database). In their study, PD-1 inhibitors (nivolumab® and pembrolizumab®) have induced the majority of cardiac disorders. Numerically, at least 80% (676/839 patients) of the total cardiac disorders had a relationship with the treatment of anti-PD-1/PD-L1 axis treatment. Eight hundred thirty-nine patients were confirmed with PD-1/PD-L1 inhibitor-related myocarditis, accounting for 15.4% of all cardiac disorders; 0.65% of all irAEs are caused by immune checkpoint inhibitor treatment (Rubio-Infante et al., 2021). Furthermore, using the same database, Salem et al. concluded similar outcomes that the incidence of anti-PD-1/PD-L1 treatment-related myocarditis was only 0.41% (84/28,909 patients). But with the combination of CTLA4 inhibitors, the myocarditis has tripled to 1.33% (32/2,412 patients) (94.6% cases in this study received PD-1/PD-L1 antibodies) (Salem et al., 2018).

Moslehi et al. reported that patients with severe myocarditis treated with immune checkpoint inhibitors showed a wide range of age at the onset, with a median of 69 years (from 20 to 90 years old), most commonly happened in melanoma and lung cancer (partially due to the selection bias in this study). The main onset is 27 days (widely from 5–155 days), and 76% occurred in the first 6 weeks of treatment (Moslehi et al., 2018). Mahmood found that the mean onset age of patients (*n* = 35) with immune myocarditis was 65 years. The mean time to onset in this study was 34 days, with 81% of cases happening within 90 days of starting the treatment (Mahmood et al., 2018). Salem et al. concluded that median onset day of myocarditis was 30 days, and most cases happened early after immune checkpoint inhibitor administration. But there was a dramatic decrease in the proportion of immune checkpoint inhibitors associated with significant cardiovascular irAEs after 3 months, reducing from about 70% to nearly 20% (Salem et al., 2018). This result was similar to what Moslehi and Mahmood have found, manifesting that the age of onset was about 65–70 years, usually owning an acute course within 1–3 months. Moslehi and Salem both confirmed that mortality was higher with a combination of anti-PD-1/PD-L1 plus anti-CTLA4 than with monotherapy (67 vs 36%, *p* = 0.008; 65.6 vs 44.4%, *p* = 0.04). In general, the results of multiple clinical studies indicate that PD-1/PD-L1 inhibitors related myocarditis is relatively rare. The age of onset is mostly 65–70 years old, and the time of onset is mostly in the early stage of administration. The age of onset and time of onset of immune myocarditis caused PD-1/PD-L1 inhibitors are summarized in Table 1.

**TABLE 1:** Summary of onset time and age of myocarditis caused by PD-1/PD-L1 inhibitors in retrospective studies.

Authors	Onset time (days)	Onset age (years)
Awadalla et al. (2020)	57 [27–122] (median, IQR)	66 ± 14 (mean ± SD)
Mahmood et al. (2018)	34 [21–75] (median, IQR)	65 ± 13 (mean ± SD)
Salem et al. (2018)	30 [18–60] (median, IQR)	66.4 ± 12.7 (mean ± SD)
Moslehi et al. (2018)	27 [5–155] (median, range)	69 [20–89] (median, range)

IOR, inter-quartile range; SD standard deviation.

In addition, we have investigated some studies to find the incidence of other cardiac diseases (shown in Table 2). Based on the fact that those studies were not all specifically designed for PD-1/PD-L1 inhibitors, we have tried to extract PD-1/PD-L1 inhibitors associated data from some reviews (Mir et al., 2018), meta-analysis (Rubio-Infante et al., 2021), RCTs, and pharmacovigilance studies (Robert et al., 2015; Antonia et al., 2017; Barlesi et al., 2018; Horn et al., 2018; Salem et al., 2018; Jain et al., 2021).

**TABLE 2:** Summary of the incidence of cardiac diseases caused by PD-1/PD-L1 inhibitors (ICIs).

Toxicity	Authors	Incidence
Myocarditis	Mir et al. (2018)	40.20%^a^
	Rubio-Infante et al. (2021)	18.84%^b^
	Barlesi et al. (2018)	1.05%^c^
	Rubio-Infante et al. (2021)	0.72%^d^
	Salem et al. (2018)	0.50%^c^
	Jain et al. (2021)	0.05%^e^
Pericardial disease or tamponade	Mir et al. (2018)	15.28%^a^
	Rubio-Infante et al. (2021)	2.12%^b^
	Salem et al. (2018)	0.36%^c^
Vasculitis	Salem et al. (2018)	0.28%^c^
Cardiomyopathy	Mir et al. (2018)	31.94%^a^
Congestive heart failure		
	Rubio-Infante et al. (2021)	0.04%^d^
Atrial fibrillation	Rubio-Infante et al. (2021)	11.54%^b^
	Jain et al. (2021)	1.92%^e^
	Antonia et al. (2017)	0.84%^c^
	Rubio-Infante et al. (2021)	0.06%^d^
Heart failure	Rubio-Infante et al. (2021)	8.45%^b^
	Jain et al. (2021)	3.56%^e^
	Barlesi et al. (2018)	1.05%^c^
	Salem et al. (2018)	0.72%^f^
	Rubio-Infante et al. (2021)	0.15%^d^
	Socinski et al. (2018)	0.25%^c^
Pericardial effusion	Rubio-Infante et al. (2021)	8.14%^b^
	Antonia et al. (2017)	0.42%^c^
	Rubio-Infante et al., 2021)	0.15%^d^
Tachycardia	Rubio-Infante et al. (2021)	6.60%^b^
	Antonia et al. (2017)	0.21%^c^
Myocardial infarction	Rubio-Infante et al. (2021)	6.55%^b^
	Jain et al. (2021)	0.90%^e^
	Salem et al. (2018)	0.53%^f^
	Antonia et al. (2017)	0.42%^c^
	Socinski et al. (2018)	0.25%^c^
Cardiac arrest	Rubio-Infante et al. (2021)	5.40%^b^
	Rubio-Infante et al. (2021)	0.02%^d^
Cardiac death or shock	Salem et al. (2018)	0.43%^f^
	Antonia et al. (2017)	0.21%^c^
Sinus tachycardia	Rubio-Infante et al. (2021)	2.23%^b^
Pericarditis	Rubio-Infante et al. (2021)	3.37%^b^
Arrhythmia	Rubio-Infante et al. (2021)	2.59%^b^
	Salem et al. (2018)	0.07%^f^
Cardiac supra-ventricular arrhythmias	Salem et al. (2018)	0.71%^f^
Atrioventricular block	Horn et al. (2018)	0.50%^c^
	Rubio-Infante et al. (2021)	0.04%^d^
Cardiac conductive disorders	Jain et al. (2021)	1.46%^e^
	Salem et al. (2018)	0.12%^f^
Endocardial disorders	Salem et al. (2018)	0.03%^f^
Cardiac valve disorders	Salem et al. (2018)	0.02%^f^
Hypertension	Socinski et al. (2018)	19.10%^c^
	Barlesi et al. (2018)	4.20%^c^
	Robert et al. (2015)	0.72%^c^
	Antonia et al. (2017)	0.21%^c^
Stroke	Jain et al. (2021)	3.71%^e^

a Incidence of disease in the patients with cardiac disease caused by PD-1/PD-L1 inhibitors.

b Incidence of disease in the patients with cardiac diseases caused by PD-1/PD-L1 inhibitors (data retrieved from the VigiAccess database).

c Incidence of disease in the patients using PD-1/PD-L1 inhibitors.

d Incidence of disease in the patients using ICI inhibitors (data retrieved from meta-analysis).

e Incidence of disease in the patients with cardiac diseases caused by PD-1/PD-L1 inhibitors (data retrieved from the International Business Machines (IBM) MarketScan Research Databases).

^f^Incidence of disease in the patients using ICI inhibitors (the proportion of CTLA4 inhibitors is 26.4%).

## 3 Clinical Characteristics

Since PD-1/PD-L1 inhibitors associated with cardiotoxicity may progress rapidly and cause hemodynamic instability, clinicians should maintain a high degree of alertness to patients receiving immunotherapy and showing symptoms of heart failure. Early detection of cardio-related symptoms is particularly important to improve the prognosis of patients. The symptoms of immune myocarditis are diverse. It can also be manifested as chest pain, palpitations, myalgia, hypotension, heart failure, complete heart block, and lower extremity edema (Frigeri et al., 2018; Yamaguchi et al., 2018; Charles et al., 2019), while some cases only manifested as non-specific symptoms such as fatigue, general malaise, ptosis, diplopia, paresis, asthenia, dyspnea, stiffness, rash, vomiting, and nausea (Gibson et al., 2016; Chen et al., 2018; Monge et al., 2018; Salem et al., 2018; Agrawal et al., 2019; Martin Huertas et al., 2019; Salem et al., 2019). Severe cases manifest major adverse cardiovascular events (MACE) as hemodynamically unstable and/or electrophysiologically unstable fulminant myocarditis, cardiogenic shock and death, cardiac arrest, and heart block, while mild cases may only have elevated cardiac markers without symptoms (Johnson et al., 2016; Escudier et al., 2017; Ganatra and Neilan, 2018; Finke et al., 2021). Mahmood has concluded in their published study that nearly half of myocarditis cases (16/35 cases) have experienced MACE (Mahmood et al., 2018). Because of the high fatality rate of myocarditis, early detection and diagnosis of myocarditis is the key to the treatment. The clinical characteristics of 29 previously reported myocarditis cases caused by PD-1/PD-L1 inhibitors are summarized in Table 3.

**TABLE 3:** Summary of reported myocarditis cases caused by PD-1/PD-L1 inhibitors.

Drugs	Dosage	Authors	Patients (Age/Sex)	Cancer Type	Onset Time	Symptoms	Diagnosis	Other irAEs	Treatment	Outcome
Nivolumab^®^	240 mg/15 days	Salem et al. (2019)	66/Female	Lung cancer	After three doses	Initial symptoms: ptosis, diplopia, painful paresis of proximal muscles Other symptoms: chest pain, elevated troponin T and NT-proBNP ECG: electrocardiographic repolarization abnormalities	CMR	Myositis	Methylprednisolone at 50 mg/day IV for 3 days, plasmapheresis, abatacept at 500 mg q2w for five doses	NR
Nivolumab^®^	NR	Martin Huertas et al. (2019)	80/Male	Kidney cancer	More than 2 months after the initial dose	Initial symptoms: asthenia, pain Other symptoms: elevated CK, troponin I, BNP ECG: atrial fibrillation, left bundle branch block	Autopsy: lymphocytes infiltration and myocardial necrosis	Myasthenia gravis	Methylprednisolone at 2 mg/kg/day IV	Death
Nivolumab^®^	3 mg/kg every 2 weeks	Charles et al. (2019)	33/Male	Hodgkin lymphoma	After the eight infusion	Symptoms: elevated troponin	CMR	Pancreatitis, dermatitis, hypothyroidism, muscular polymyositis	Mycophenolate mofetil, steroids at 1 to 2 mg/kg, IGI	Death
						ECG: complete heart block				
Nivolumab^®^	240 mg IV	Agrawal et al. (2019)	67/Male	Melanoma	Prior to fourth cycle	Initial symptoms: chest pain and palpitations Other symptoms: elevated troponin I ECG: atrial fibrillation ECHO: bi-ventricular dilatation, reduced right ventricular systolic function, LVEF of 55%	CMR: myocardial edema (elevated regional T2 ratio and early gadolinium enhancement ratio)	Optic neuritis	Methylprednisolone 1 g/d IV for 3 days, prednisone 80 mg BID for 5 days then tapering, infliximab, oral corticosteroids	Symptoms improved
Nivolumab^®^	240 mg IV	Agrawal et al. (2019)	65/Male	laryngeal cancer	Six days after the first dose	Initial symptom: dyspnea Other symptoms: edema, hypotention of systolic blood pressures ECG: bradycardia ECHO: global hypokinesis, LVEF of 25–30%, LVEF back to 50% after steroid treatment	Lake Louise Criteria (Friedrich et al., 2009)	NR	Methylprednisolone at 1 g/d for 3 days IV, diuretics, ACEI, carvedilol, prednisone OP, ATG	Death
						CMR: edema and early gadolinium enhancement in T2				
Nivolumab^®^	1 mg/kg every 2 weeks	Monge et al. (2018)	79/Male	Prostate cancer	Eight weeks later	Initial symptoms: blurred vision, pain, and stiffness in the upper back Other symptoms: elevated troponin I, CK, CK-MB, and ProBNP ECG: atrial fibrillation, left anterior fascicular block ECHO: LVEF of 65%, increased pulmonary artery pressure (45 mmHg)	CMR: patches of LGE	NR	Methylprednisolone 1 mg/kg/day IV, prednisone taper	Clinical recovery
Nivolumab^®^	2 mg/kg	Yamaguchi et al. (2018)	60/Male	Melanoma	After 13 cycles	Initial symptoms: fatigue and fever Other symptoms: hypotention, tachycardia, elevated PAWP of 23 mmHg ECG: ST-segment elevation in V4–6, leads II, III, and aVF ECHO: LVEF of 15%, myocardial edema, hypokinesis, back to56% after treatment	Myocardial biopsy: lymphocytic infiltration	NR	Prednisolone at 1,000 mg/d for 3 days IV, IGI at 50 g/d for 2 days IV, ECMO, IABP	Symptoms improved
Nivolumab^®^	NR	Frigeri et al. (2018)	76/Female	Lung cancer	After 7 biweekly administrations	Initial symptoms: heart failure (bibasilar pulmonary rales and lower limb edema, elevated NT-proBNP and troponin) ECHO: LVEF at 15% initially, droped to less than 10%, finally back to 30% after treatment CMR: diffuse delayed myocardial contrast enhancement	According to clinical symptoms	NR	Methylprednisolone 5 mg/kg/d and three doses of infliximab 5 mg/kg, ECMO, IABP, plasmapheresis, cardioverter-defibrillator implanted	Symptoms improved
Nivolumab^®^	3 mg/kg	Chen et al. (2018)	43/Male	Thymoma	10 days later	Initial symptoms: chest discomfort, fatigue, myalgias of lower limbs Other symptoms: chest pain, dyspnea, rash, diplopia or dysphagia, elevated troponin I, CK, CK-MB, NT-Pro BNP and myoglobin, cardiac arrest, hypotention, multisystem organ failure ECG: prolongation of the PR interval, RBBB, second degree of AV block, extensive ST-elevation ( lead I, AVL, AVR, V1–V5) ECHO: LVEF of 49%, thickened interventricular septum, gradual hypokinesis with LVEF down to 40% Cause of death: multisystem organ failure	Autopsy: interstitial edema accompanied by lymphocytic infiltrates	Rhabdomyolysis	IGI at 300 mg/kg for 4 days IV, methylprednisolone 1 g/day for 3 days followed by 500 mg/day for 4 days then 60 mg/day, IABP, hemodialysis, temporary pacemaker implanted	Death
Nivolumab^®^	2 mg/kg every 3 weeks	Tadokoro et al., 2016)	69/Female	Melanoma	Two weeks after the last dose	Initial symptoms: malaise and palpitation Other symptoms: tachycardia (110 beats per minute), ST-segment elevation (leads II, III, and aVF), elevated troponin T, CK and CK-MB ECHO: diffuse hypokinesis (LVEF of 30%), LVEF back to 55% after treatment	Myocardial biopsy: lymphocytic infiltration (a predominance of CD8-positive, PD-1-negative T cells)	NR	Prednisolone at 2 mg/kg initially IV, sequently tapered at 5 mg per week	Clinical recovery
Nivolumab^®^	3 mg/kg	Matson et al., 2018)	55/Male	Lung cancer	About 46 days after the first administration	Initial symptom: lethargy, shortness of breath Other symptoms: diabetic ketoacidosis, ventricular tachycardia, heart failure, elevated troponin I, multi-organ failure Cause of death: cardiogenic shock caused by right-sided heart failure and multi-organ failure	Autopsy: T-cell infiltration	Diabetes mellitus	NR	Death
Nivolumab^®^	3 mg/kg	Mir et al., 2018)	59/Female	Renal cell carcinoma	About 4 weeks after the first dose	Initial symptom: complete heart block Other symptoms: chest pain, elevated troponin T Coronary angiography: wall motion abnormalities Cause of death: ventricular fibrillation	Autopsy: diffuse infiltration of lymphocytes, macrophages, and giant cells	NR	Pacemaker implantation	Death
Nivolumab^®^	NR	Gibson et al., 2016)	68/Female	Lung cancer	One week after the second dose	Initial symptoms: nausea and vomiting Other symptoms: elevated AST, ALT, ALP, CK, CK-MB ECG: RBBB, left posterior fascicular block, tachycardia, multiple ectopic beats	Clinical symptoms and ICIs treatment	NR	Methylprednisolone, amiodarone infusion	Clinical recovery
Nivolumab^®^	3 mg/kg every 2 weeks	Semper et al., 2016)	75/Male	Lung cancer	Three days after the 9th cycle	Initial symptoms: dyspnea and chest pain Other symptoms: elevated troponin T, CK ECG: atrial fibrillation, complete right bundlebranch block, left anterior fascicular block	CMR	NR	ACE inhibitors, beta blockers, diuretics, prednisolone at 1 mg/kg/day	Clinical recovery
Pembrolizumab^®^	After 4 circles of ipilimumab^®^ and pembrolizumab^®^ 2 mg/kg every week	Läubli et al., 2015)	73/Male	Melanoma	After 5 circles of pembrolizumab^®^	Initial symptoms: dyspnea, bilateral rales and lower leg edema Other symptoms: elevated troponin T, NT-proBNP and D-Dimer ECG: tachycardia, incomplete right bundle branch block, left axis deviation, a *de novo* minor ST-Segment depression in V4-V6 ECHO: discrete septal hypokinesia and first-degree aortic valve insufficiency CMR: LVEF of 49%, LGE	Myocardial biopsy: infiltration predominantly CD8-positive T cells	NR	Candesartan, beta-blocker, diuretics, spironolactone, torsemide, and prednisone 2 mg/kg IV	Clinical recovery
Pembrolizumab^®^	NR	Esfahani et al. (2019)	71/Female	Melanoma	After 2nd cycle of treatment	Initial symptoms: shortness of breath and ptosis Other symptoms: troponins I and T, respiratory failure ECG: ventricular tachycardias, fast paroxysmal atrial fibrillation, cardiac pauses	CMR	Myositis–myasthenia gravis overlap syndrome	Methylprednisolone at 1 g/day for 3 days IV, then mycophenolate at 2 mg/kg/day, mycophenolate mofetil at 2 g/day, plasmapheresis, rituximab at 375 mg/m^2^ IV, alemtuzumab at 30 mg	Completely recovered
Pembrolizumab^®^	200 mg	Agrawal et al. (2019)	73/Male	Malignant Mesothelioma	32 days later	Initial symptoms: dyspnea, edema and fatigue Other symptoms: elevated troponin I, CK and CK-MB; ECG: RBBB, LBBB, episodes of asystole ECHO: LVEF of 50–60%, mild LVH, right and left atrial enlargement	According to atrial-ventricular block with elevated troponin	Myasthenia crisis	Prednisolone 60 mg/day OP, permanent pacemaker implantation, IGI, lasmapheresis, ACEi, beta blocker, furosemide	Death
Pembrolizumab^®^	2 mg/kg every 3 weeks	Agrawal et al. (2019)	89/Male	Melanoma	12 days after the first dose	Initial symptoms: weakness, myalgias, dyspnea Other symptoms: elevated troponin I, CK, CK-MB, BNP, ALT, AST ECG: RBBB, LBBB, episodes of asystolem, complete heart block ECHO: right ventricular dilation, Mobitz I AV block cause of death: infection and multi-organ failure	According to clinical symptoms and history of ICIs treatment	NR	Methylprednisolone at 1 g/day IV, prednisone 60 mg twice daily OP, ATG 125 mg daily, trans-venous pacing wire placed	Death
Durvalumab^®^	1.125 mg/kg	Bockstahler et al. (2020)	75/Male	Lung cancer	2 months after the first dose	Initial symptoms: dyspnea and chest pain	EMB: ICI-related myocarditis	NR	Prednisolone 160 mg/d	Symptoms improved
						Other symptoms: elevated troponin T, CK				
						ECG: atrial fibrillation, complete right bundlebranch block, left anterior fascicular block				
Nivolumab^®^ and Ipilimumab^®^	ipilimuma®b at 3 mg/kg, nivolumab^®^ at 1 mg/kg given every 3 weeks for 4 cycles	Khoury et al. (2019)	68/Male	Melanoma	Two weeks after the second dose	Initial symptoms: shortness of breath, skin rash, and double vision Other symptoms: elevated CPK (back to normal baseline), troponin I ECG: tachycardia Cause of death: sudden cardiac death	Clinical and biochemical findings	Hypophysitis, oculomotor neuritis	Solumedrol at 1 g/day divided into four doses for 3 days IV, prednisone 2 mg/kg and decreasing the dose daily by 7.5%	Death
Nivolumab^®^ and Ipilimumab^®^	NR	Ganatra and Neilan (2018)	41/Female	Melanoma	6 days after four cycles	Initial symptoms: dyspnea, tachycardia Other symptoms: mildly elevated troponin I ECG: sinus tachycardia ECHO: LVEF of 15% CT: cardiomegaly and pulmonary congestion CMR: T2 hyper-intensity and LVEF of 12%	EMB: lymphocytic infiltrate (CD-3 and CD-8 T cells) and mild interstitial fibrosis	NR	Methylprednisolone at 1 g/day for 3 days IV and neurohormonal antagonists	Symptoms improved
Nivolumab^®^ and Ipilimumab^®^	Every 3 weeks	Thibault et al. (2018)	52/Male	Renal cell carcinoma	Three circles later	Initial symptoms: asymptomatic with mild troponin I	CMR	NR	Combination of ICIs transferred to nivolumab^®^ alone with beta blocker therapy	No subsequent clinical event
Nivolumab^®^ and Ipilimumab^®^	NR	Tajmir-Riahi et al. (2018)	72/Male	Melanoma	After the 10th therapy	Initial symptoms: dyspnea, edema of the legs with pleural effusion and ascites ECHO: LVEF down from 48–50%–15% (back to 35–40% after useage of steroid) Cause of death: cardiac arrest	Myocardial biopsy: absence of FOXP3-positive cells and lymphocytic infiltration	Hypophysitis, thyroiditis	Prednisolone at 1 mg/kg/day, diuretic	Death
Nivolumab^®^ and Ipilimumab^®^	Ipilimumab^®^ (3 mg/kg) plus nivolumab^®^ (1 mg/kg)	Norwood et al. (2017)	49/Female	Melanoma	2 weeks after the first dose	Initial symptom: nausea; Other symptoms: elevated cTnI, CK-MB and total creatine kinase	EMB: collagen deposition and inflammatory cells (CD3^+^ T cells and CD68^+^ macrophages)	Thyroiditis	Methylprednisolone at 125 mg/day, IV; prednisone at 1 mg/kg daily and tapered over 1 month, OP; IGI 400 mg/kg/day for 2 days, IV	Clinical recovery
Nivolumab^®^ and Ipilimumab^®^	Ipilimumab^®^ (3 mg/kg) plus nivolumab^®^ (1 mg/kg)	Johnson et al. (2016)	65/Female	Melanoma	12 days after the first doses	Initial symptom: atypical chest pain, dyspnea and fatigue Other symptoms: elevated CPK, CK-MB, troponin I Cause of death: ventricular tachycardia, multisystem organ failure ECG: PR prolongation, intraventricular conduction delay, complete heart block	Autopsy: lymphocytic infiltration in myocardium	NR	Methylprednisolone at 2 mg/kg/day, IV	Death
Nivolumab^®^ and Ipilimumab^®^	Ipilimumab^®^ (3 mg/kg) plus nivolumab^®^ (1 mg/kg)	Johnson et al. (2016)	63/Male	Melanoma	15 days after the first doses	Cause of death: cardiac arrest ECG: profound ST-segment depression, intraventricular conduction delay ECHO: LEVF of 50%	Post-mortem histopathology: T-cell and macrophage infiltration	NR	Methylprednisolone 1 g/kg/day for 4 days, IV; infliximab 5 mg/kg, IV	Death
Nivolumab^®^ and Ipilimumab^®^	Ipilimumab^®^ (3 mg/kg) plus nivolumab^®^ (1 mg/kg, 4 infusions followed by 3 mg/kg every 2 weeks)	Heinzerling et al. (2016)	72/Male	Melanoma	After 3 infusions of ICIs combination	Initial symptoms: dyspnea, peripheral edema and anasarca ECHO: LEVF from 50 to 15%, back to 40% after steroids treatment	Cardiac biopsy: lymphocytes and interstitial fibrosis	Thyroiditis and hypophysitis	Anti-immunity: corticosteroids were initiated at 1 mg/kg orally; Other treatment: furosemide, spironolactone 25 mg/day, ramipril 2 × 5 mg/day, metoprolol 2 × 47.5 mg/day	Symptoms improved
Nivolumab^®^ and Ipilimumab^®^	NA	Mehta et al. (2016)	68/Female	Melanoma	After the 2nd dose	Initial symptoms: left arm pain, substernal chest pain, fever, and malaise Other symptoms: elevated troponin T and CK-MB CMR: subepicardial and mid wall delayed hyperenhancement involving the lateral wall	Clinical symptoms and usage of ICIs	NR	Methylprednisolone at 1 mg/kg IV, then 7-week taper of oral prednisone	Clinical recovery
Atezolizumab^®^ and Nivolumab^®^	Atezolizumab^®^ (1,000 mg) nivolumab^®^ (3 mg/kg/2 weeks)	Liu et al. (2019)	61/Female	Lung cancer	Three days after the first dose of atezolizumab, about 18 weeks after the first dose of nivolumab^®^	Initial symptoms: dyspnea, fatigue Cause of death: cardiac arrest Symptoms: elevated CK-MB, troponin I, and NT-proBNP Chest X-ray: right lung consolidation ECG: sinus tachycardia	Autopsy: infiltration of macrophages, CD8^+^ T cells, and focal fibrosis	NR	Methylprednisolone at 5 mg/kg/day IV, mycophenolate mofetil 1,000 mg/day OP	Deterioration
										
										
										
										

NR, no report; NT-proBNP, N-terminal pro-brain natriuretic peptide; BNP, brain natriuretic peptide; ECG, electrocardiogram; CK, creatine kinase; CK-MB, creatine kinase-myocardial band; ATG, anti-thymocyte globulin; CMR, cardiovascular magnetic resonance; ECHO, echocardiogram; LVEF, left ventricular ejection fraction; ACEI, angiotensin-converting enzyme inhibitors; PAWP, pulmonary artery wedge pressure; LGE, late gadolinium enhancement; ECMO, extracorporeal membrane oxygenation; IABR, intra-aortic balloon pump; LBBB, left bundle branch block; RBBB, right bundle branch block; LVH, left ventricular hypertrophy.

## 4 Diagnosis

The incidence of immune myocarditis is still unclear, which has ranged from 0.39 to 0.8% (Johnson et al., 2016; Salem et al., 2018). Ganatra et al. assumed that the incidence of true immune myocarditis may be underestimated due to multiple factors, including lack of specific cardio-related symptoms, possibility of overlap with other cardiovascular diseases, non-standard, and non-specific diagnostic measurements (Ganatra and Neilan, 2018). The common clinical diagnostic methods for myocarditis include symptoms, electrocardiogram, biomarkers (creatine kinase isoenzyme, troponin I, brain natriuretic peptide, etc.), and cardiac imaging (echocardiography and cardiac magnetic resonance). Endomyocardial biopsy has been regarded as the diagnostic gold standard for PD-1/PD-L1 inhibitor-associated myocarditis.

### 4.1 Troponin

According to the characteristics of troponins, the type of cardiac troponin I and T are the new isoforms that are present in adult cardiac tissues (Yu et al., 2021). About 46–97% of patients with immune myocarditis have increased serum troponin levels (Escudier et al., 2017; Mahmood et al., 2018; Mir et al., 2018; Awadalla et al., 2020; Finke et al., 2021). In Awadalla’s study, the troponin levels of patients with immune checkpoint inhibitor treatment were elevated, with a median value of 0.85 ng/dl. But the troponin levels were <0.01 ng/dl in the control group (*p* < 0.001). Mahmood advocated that troponin T of ≥1.5 ng/ml may be related to a fourfold increased risk of MACE caused by immune myocarditis. Cardiac troponin T (cTnT) is also increased in patients with myositis but cardiac troponin I (cardiac troponin I, cTnI) is more specific to myocardial injury (Lilleker et al., 2018). Cardiac tissue damage, like cell death or necrosis with myofilament injuries, will inevitably cause cTn elevation. Meanwhile, the severity of cardiac damage can be reflected by the level of increased cTn (Bass et al., 2010). Mir and Heinzerling have reported that some cases with PD-1/PD-L1 inhibitor immunotherapy-related heart failure, cardiomyopathy, myocarditis, and myocardial fibrosis may manifest normal troponin levels (Heinzerling et al., 2016; Mir et al., 2018). Unfortunately, there was no study that can tell us that the specific troponin level can play a role in predicting PD-1/PD-L1 inhibitor-associated myocarditis.

### 4.2 Brain Natriuretic Peptide

Based on the present study, serum brain natriuretic peptide (BNP) or N-terminal-pro brain natriuretic peptide (NT-pro BNP) levels increased in 65.71–88% of patients with immune checkpoint inhibitor-related myocarditis (Mahmood et al., 2018; Awadalla et al., 2020). Escudier reported 100% patients with elevated BNP or NT-pro BNP in their study with a limited sample size (*n* = 14, the BNP or NT-pro BNP information can be obtained from 14 out of 30 patients) (Escudier et al., 2017). The ventricular expansion and volume overload can activate the natriuretic peptide system to promote the secretion of BNP. BNP is a specific biomarker reflecting the pressure or overload. BNP can indirectly tell us the function of the left ventricular (LV) strain in patients with heart failure or myocarditis (Yu et al., 2021). All the pathogenesis which can lead to dysfunction of LV can also result in elevated BNP levels. By the way, elevated serum BNP in cancer patients can also be caused by cancer-related chronic inflammation (Bando et al., 2017). In many different cardiovascular conditions, like myocarditis, BNP and NT-proBNP are sensitive biomarkers in predicting heart failure (Elamm et al., 2012). In view of the fact that BNP is a biological marker of left ventricular systolic function, we propose a hypothesis whether BNP can be used to predict the existence of subclinical functional and morphological damage of the left ventricle in cancer patients with PD-1/PD-L1 inhibitors.

### 4.3 Electrocardiogram

Based on the universality of the electrocardiogram (ECG) examination, Ganatra suggested that the ECG should be routinely performed for suspected immune myocarditis (Ganatra and Neilan, 2018). ECG performances in PD-1/PD-L1 inhibitor-associated myocarditis cases were neither specific nor sensitive, such as sinus tachycardia, QRS/QT prolongation, conduction abnormalities, diffuse T-wave inversion, Q waves, ventricular arrhythmias, non-specific ST-segment changes, and local or diffuse ST-T elevation like the manifestation of acute coronary syndrome and conduction disorders (Johnson et al., 2016; Ammirati et al., 2017). It is difficult to make a diagnosis of myocarditis only relying on ECG. But given its availability and simplicity, it should be the first-line test for cancer patients with cardiac symptoms.

### 4.4 Echocardiogram

Echocardiogram has been considered as a useful tool for assessing the cardiac function and ruling out other cardiovascular diseases, with the limitation that cannot manifest tissue characteristics and detect small myocardial abnormalities. Decreased left ventricular ejection fraction (LVEF), abnormal left ventricular internal dimensions in diastole, and abnormal motion of the ventricular wall can be manifested by echocardiogram in PD-1/PD-L1 inhibitor-related myocarditis cases. About 40–49% of patients have decreased left ventricular systolic function (Escudier et al., 2017; Mahmood et al., 2018; Awadalla et al., 2020). Escudier reported that nearly half of the patients had a significant decrease in LVEF (≤35%) (Escudier et al., 2017). Awadalla et al. showed that cases with decreased LVEF (median LVEF was 45%) had more possibility to develop MACE compared to the normal myocarditis patients with normal LVEF (median LVEF was 55%) (Awadalla et al., 2020). Different from the traditional fulminant myocarditis with a significant decrease in LVEF (Ammirati et al., 2017; Zhang et al., 2020), 38% of patients with fulminant myocarditis caused by immune checkpoint inhibitors had normal LVEF (Mahmood et al., 2018). Therefore, it may be not safe to rule out severe PD-1/PD-L1 inhibitor fulminant myocarditis according to the normal LVEF in echocardiogram.

### 4.5 Cardiovascular Magnetic Resonance

Cardiovascular magnetic resonance (CMR) can provide better spatial resolution and tissue specificity than echocardiogram; show myocardial edema, necrosis, and scar formation caused by myocarditis; and can manifest left ventricular systolic function. Myocardial necrosis and fibrosis are manifested by late gadolinium enhancement (LGE) (Frigeri et al., 2018; Zhang et al., 2020; Finke et al., 2021). Although CMR has a sensitivity of 89% and a specificity of 96% in the diagnosis of other forms of myocarditis (Yang and Asnani, 2018), the incidence of LGE happened in immune checkpoint inhibitor-related myocarditis ranging from 23 to 48% (Escudier et al., 2017; Zhang et al., 2020). Escudier found only 3 cases with LGE out of 13 patients, making the incidence less convinced. Zhang et al. concluded that LGE increased from 21.6 to 72.0% with prolonged hospitalization. We wonder whether LGE has relationship with the time when CMR was completed, so the incidence of LGE need to be confirmed by randomized control trials with large sample size.

### 4.6 Endomyocardial Biopsy

Endomyocardial biopsy (EMB) is the gold standard for diagnosing myocarditis (Guglin and Nallamshetty, 2012; Sagar et al., 2012). The pathological feature of PD-1/PD-L1 inhibitors related was T-cell infiltration. Mostly, the infiltrating cells were CD8^+^ T and CD4^+^ T cells. Macrophages were also common. Some cases may have eosinophil, giant cells, and neutrophils infiltration (Heinzerling et al., 2016; Johnson et al., 2016; Mahmood et al., 2018; Champion and Stone, 2020). T-lymphocyte infiltration can be found in the atria, ventricles, cardiac conduction system, and interventricular septum. In addition, necrosis, edema, or fibrosis of the myocardium can also be found in the cardiac tissues with immune myocarditis (Heinzerling et al., 2016; Johnson et al., 2016; Norwood et al., 2017; Matson et al., 2018; Champion and Stone, 2020; Sobol et al., 2020). Although EMB is the most accurate diagnostic method for PD-1/PD-L1 inhibitor-related myocarditis, the operation was really accompanied by certain risks, such as transient arrhythmia, pericardial tamponade, and myocardial perforation (Yang and Asnani, 2018). Champion and Stone have proclaimed that the myocyte injury caused by myocarditis was in discrete foci, with no case with diffuse myocarditis throughout the whole specimen (Champion and Stone, 2020). So under this setting, EMB may provide us the false-negative results (Agrawal et al., 2019).

## 5 Pathogenesis

### 5.1 Autoimmunity-Related T-Lymphocyte Infiltration

As mentioned above, activated T cells can express one kind of immune-inhibitory checkpoint on their surface named PD-1. When PD-1 binds to its ligands, T cells lose their functional abilities. This interaction is a kind of significantly important immunologic tolerance mechanism in the peripheral tissues and at sites of ongoing inflammation (Fife and Bluestone, 2008). Under this circumstance, even auto-activated T cells flow into the myocardium; PD-1 signaling can restrict T cells, making immune homeostasis (Yi et al., 2021). PD-L1 has been already confirmed to express on the cardiac tissue (Dong et al., 1999; Freeman et al., 2000; Yamaguchi et al., 2018). Douglas and others have carried out T-cell receptor next-generation sequencing (CDR3 region, the antigen-binding portion of the T-cell receptor beta chain), indicating that patients with PD-1/PD-L1 inhibitors related myocarditis have their own high-frequency T-cell receptor sequences among cardiac, skeletal muscle, and tumor tissue (Johnson et al., 2016). PD-1/PD-L1 monoclonal antibodies block PD-1 or its ligand, resulting in invigoration of these quiescent T cells again. That was helpful for the T cells to eliminate the tumor cells, at the same time providing activated T cells a possibility to non-selectively attack the normal cells. The autoimmune phenomenon may account for the immune-related adverse events (irAEs) (Cousin and Italiano, 2016). Carolyn and Douglas have previously reported some cases of myocarditis induced by PD-1/PD-L1 inhibitor treatment, with strong evidence of lymphocytic infiltration when the PD-1\PD-L1 axis was cut (Johnson et al., 2016; Glass and Mitchell, 2017), which was consistent with the histological examination in myocarditis cases (Tarrio et al., 2012; Heinzerling et al., 2016; Chen et al., 2018; Ganatra and Neilan, 2018; Mir et al., 2018; Yamaguchi et al., 2018; Martin Huertas et al., 2019). Ji et al. (2019) found mononuclear cell infiltration in myocardium with myocarditis in monkeys, with a large proportion of T cells (CD3^+^, CD4^+^, and CD8^+^). Heinzerling have found the same pathological manifestation in their cases (Heinzerling et al., 2016). Hyperproliferation of T cells happened in the monkey model with PD-1 inhibition treatment. Wang et al. have found that MRL-PD-1^−/−^ mice had more possibility to develop fatal myocarditis with antibodies against cardiac myosin. They have also investigated T cells in the hearts of MRL-PD-1^−/−^ mice but not in the spleen and lymph nodes were activated, which suggested that antigen-specific autoimmune response may mediate the myocarditis (Wang et al., 2010). Muath and his colleagues have investigated PD-1/PD-L1 expression in human transplant hearts. In the rejecting hearts caused by vasculopathy, PD-L1 expression was nearly absent in the myocardium, instead PD-1 expression was high. Meanwhile, PD-1 and PD-L1 owned similar and moderate expression levels in non-rejection allografts. By the way, PD-1/PD-L1 inhibitor agents blocking the specific immune checkpoint will achieve the disproportionate expression level between PD-1 and PD-L1, making the combination of PD-1/PD-L1 impossible with the unmatched numbers. Muath provided us a new perspective that the different expression levels between PD-1/PD-L1 (increased PD-1, decreased PD-L1) may be the trigger to cause the autoimmune responses in the myocardium (Bishawi et al., 2021).

### 5.2 Disorder of Regulatory T-Cell Homeostasis

Regulatory T cells (Tregs) suppress autoimmunity mainly through inhibitory receptors, like CTLA4 and PD-1. And the deficiency of these inhibitory receptors can result in the dysfunction of Tregs. PD-L1 has been testified to induce and maintain Tregs to inhibit T-cell function (Francisco et al., 2009; Zhang et al., 2016). PD-1 absence with forkhead box P3 (FoxP3) insufficiency can cause generation of ex-FoxP3 (FoxP3 expression) T cells with pro-inflammatory properties, accompany the expansion of effector T cells (Teffs) and memory T cells (Tregs), thereby promoting the autoimmune destruction of target tissues (Zhang et al., 2016; Tajmir-Riahi et al., 2018). Genetic or environmental disturbances that influence both PD-1 and Foxp3 will lead to the disruption of T-cell homeostasis, causing lethal autoimmune disease (Zhang et al., 2016). Alissafi et al. have reported that Foxp3^+^ Tregs may suppress the autoimmune via inhabitation of autophagic machinery of dendritic cells in the CTLA4-mediated pathway. And anti-CTLA4 treatment can disrupt the interaction of DCs and Tregs, making autoimmune aggravation (Alissafi et al., 2017). Foxp3^+^ Tregs are the fundamental stone of immune homeostasis. PD-1/PD-L1 inhibitors disturb Tregs, resulting in imbalance between Teffs and Tregs in normal tissues, which may be the origin of irAEs, consistent with the myocardial biopsy result found by Laubli which stated that only a few Foxp3^+^ Tregs were found in myocardial tissue with signs of apoptosis (Läubli et al., 2015; Kumar et al., 2020).

### 5.3 Cytokines

Rodig et al. have found that tumor necrosis factor-α (TNF-α) and interferon-γ (IFN-γ) can stimulate PD-L1 expressed on endothelial cells, with the inhibition of T cells (Rodig et al., 2003). Grabie et al. have also testified that myocarditis induced by cytotoxic T-lymphocyte was accompanied by the upregulation of PD-L1 expressed on cardio-endothelia. And the PD-L1 was upregulated by the stimulation of interferon-γ. So anti-IFN-γ treatment can lead to high expression of PD-L1, finally causing aggravation of myocarditis (Grabie et al., 2007). Meanwhile, the PD-L1/2^−/−^ mice were more susceptible to severe myocarditis. And the same outcomes can be obtained via PD-L1 blocking treatment (Rodig et al., 2003). Seko et al. have also found that IFN-γ secreted from infiltrating lymphocytes can induce PD-L1 expression in myocarditis. They also testified that anti-PD-1 monoclonal antibodies can increase the level of IFN-γ, Fas ligand, pore-forming protein (PFP), which have been confirmed to promote the inflammatory process. IFN-γ, Fas ligand, and PFP potentially become the activator for the infiltrating lymphocytes in myocarditis (Seko et al., 2007). Zheng et al. (2018) have confirmed that the overexpression of fibrinogen-like protein-2 (FGL2) can significantly reduce PD-1 and aggravated cardiac function in mice with immune-related myocarditis. And they also found that forkhead box P3 (Foxp3) significantly increased in those mouse models, which was considered to collaborate with PD-1 to maintain and regulate the function of Tregs (Zhang et al., 2016). Finke has found that upregulation of the IFN-γ pathway can assemble the NLRP3 inflammasome (Finke et al., 2021), which has been testified to increase the number of infiltrating CD8^+^ T cells (Theivanthiran et al., 2020). Cytokines are reservoirs for mechanism research, and the levels of various cytokines can regulate the expression of PD-1 or PD-L1, thereby affecting the PD-1/PD-L1 axis.

### 5.4 Synergistic Effect of PD-1/PD-L1 and CTLA4

**FIGURE 2:**
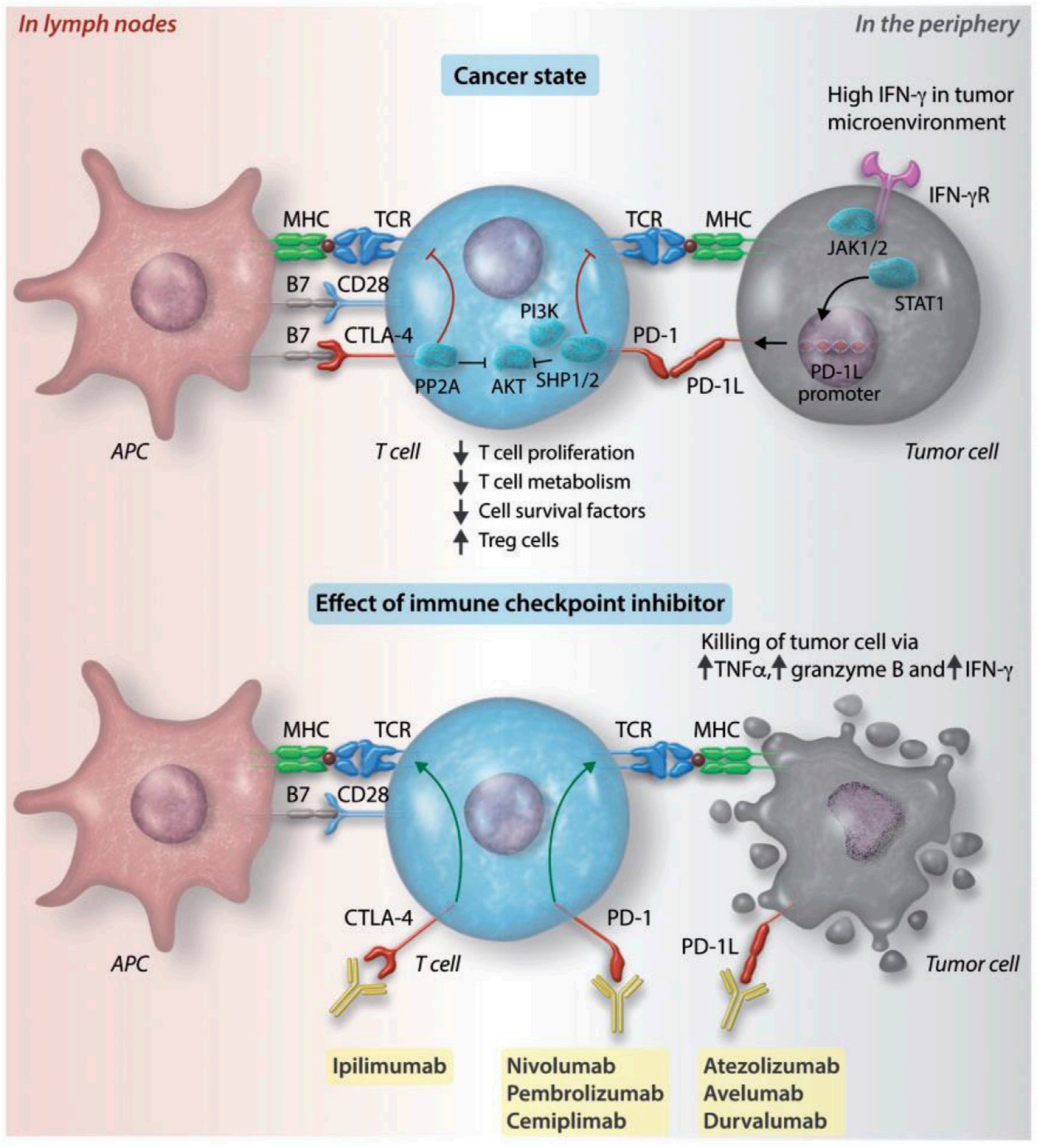
Attenuation and activation of T lymphocytes via CTLA4 and PD-1/PD-L1 axes. APCs can capture neoantigens and present to T lymphocytes on MHC molecules. The activated T cells, receiving the neoantigens, will migrate to the tumor bed from lymph nodes. The activation of T cells can be accelerated by costimulatory signals via the combination of B7 with CD28. The attenuation of T cells is managed by inhibitory axes, which are CTLA4/B7 and PD-1/PD-L1. The tumor cells can recognize the IFN-γ via the IFN-γ receptor, which promotes the expression of PD-L1 on their surfaces. PD-L1 can help tumor cells to escape the attack of T cells. The monoclonal antibodies of those immune checkpoints, like CTLA4 (ipilimumab^®^), PD-L1 (atezolizumab^®^, avelumab^®^, and durvalumab^®^), and PD-1 (nivolumab^®^, pembrolizumab^®^, and cemiplimab^®^), can stop the attenuation of T cells, making activated T cells to attack tumor cells. Modified and reprinted from Han J. R. et al. (2019). Copyright ^©^ The Author(s). 2019. Reproduction with permission from The Author(s). 2019 Open Access.

APCs will capture the neoantigens that are released by tumor cells. Through the major histocompatibility complex I (MHC I) molecule/TCR signal pathway, APCs transfer the peptides to CD8^+^ cytotoxic T cells. APCs can also present peptides through the MHC II pathway to CD4^+^ T helper cells. T cells activation relying on TCR signaling needs the help of CD28, which is costimulatory signals expressed on the surface of T cells. Upregulated T-lymphocyte-associated antigen 4 (CTLA4) on the surface of activated T cells, which may compete with CD28 for binding to CD80 and/or CD86. The conjunction of CTLA4 with CD80 or CD86 results in attenuation of T cells. So the CTLA4 checkpoint inhibitor can enhance immune responses against tumor cells that can express neoantigen, leading to tumor cells being killed (Tarrio et al., 2012; Grabie et al., 2007; Varricchi et al., 2017). Spencer has testified that there was a dosage-dependent genetic and functional interaction between the T cell with genes Ctla4 and Pdcd1. Compared to Ctla4^+/+^ Pdcd1^−/−^ mice, the Ctla4^+/−^ Pdcd1^−/−^ mice have increased immune infiltration and cardiomegaly, confirming the gene dosage of Ctla4 and Pdcd1 together defining the functional up-limit of T cells (Wei et al., 2021). It has been testified that CTLA4 and PD-1 can cooperate to attenuate T-cell activation, with cellular and molecular regulation (Chen and Flies, 2013; Wei et al., 2018; Chemnitz et al., 2004). Therefore, T-cell hyperfunction caused by monoclonal antibody treatment of CTLA4 and PD-1 is an indisputable fact. And the blockages of PD-1/PD-L1 and CTLA4 can synergistically enhance each other. The attenuation and activation of T lymphocytes via CTLA4 and PD-1/PD-L1 axes are demonstrated in Figure 2. In clinical studies, it has been proved that mortality was higher with a combination of anti-PD-1/PD-L1 plus anti-CTLA4 than with monotherapy (67 vs 36%, *p* = 0.008) (Moslehi et al., 2018; Wei et al., 2021).

### 5.5 Macrophage-Mediated Inflammatory Response

Macrophage has been proved to play a vital role in controlling inflammation. Its subtypes M1 macrophages can secrete cytokines to accelerate inflammatory responses, while M2 macrophages suppress inflammation and promote scar formation (Shivshankar et al., 2014). Hu et al. manifested that human kallikrein can reduce cardiac fibrosis related to cardiac damage or dysfunction not only by reducing, gathering, or trapping the activated M2 macrophages but also through decreased shift of macrophages to M2 (Hu D. et al., 2019). The CD68^+^ macrophages have been found in the PD-1/PD-L1 inhibitors related case (Johnson et al., 2016; Norwood et al., 2017; Mir et al., 2018; Liu et al., 2019). Xia et al. have testified that cardio-injury caused by PD-1/PD-L1 inhibitors may be explained via exosomes that derived from PD-1 inhibitor-treated macrophages (Exosome^PD−1^ inhibitor), which can lead to cardiomyocyte senescence (damage or dysfunction). In their murine models, Xia found a significant decrease in the left ventricular ejection fraction and fractional shortening in the PD-1 inhibitor group by echocardiography. More myocardial cells stuck in the G0/G1-phase were found in the PD-1 inhibitor group and a higher percentage of senescence-associated β-galactosidase (SA-β-gal) staining positive cells. On the contrary, telomere length and telomerase activity-β decreased. When miR-34a-5p inhibition was adopted in PD-1 inhibitor-treated macrophages, the percentage of G0/G1-phase cells and the number of SA-β-gal-positive cells decreased, while accompanying the recovery of the telomere length and telomerase activity. Finally, they hypothesized that Exosome^PD−1^ inhibitor may exert a pro-senescent effect through modulating the miR-34a-5p/PNUTS signaling pathway (Xia et al., 2020a). Xia et al. (2020b) have also found that the PD-1 inhibitor may promote M1 macrophage polarization and cardiac injury by modulating the miR-34a/KLF4 (Krüppel-like factor 4) signaling pathway.

Champion and Stone (2020) have also demonstrated that more CD68^+^ macrophages were found in high-grade myocarditis cases (>50 CD3^+^ cells/high power field).

Ji et al. (2019) found mononuclear cell infiltration in myocardium with myocarditis in monkeys, with a large proportion of T cells (CD3^+^, CD4^+^, and CD8^+^) and a lower number of CD68^+^ macrophages.

## 6 Treatment

The treatment of PD-1/PD-L1 inhibitors related to adverse cardiac events mainly includes symptomatic and causal treatment. If the patient has symptoms of chronic heart failure, β-blockers and angiotensin-converting enzyme inhibitor/angiotensin II receptor antagonists are used for treatment. The main causal treatment is to suppress the over-activated T lymphocytes. The first-line immunosuppressant is the first choice for high-dose corticosteroids. The National Comprehensive Cancer Network (NCCN) has summarized the grades of cardiovascular adverse events that may be related to immune checkpoint inhibitor treatment. For grade 2, 3, and 4 cardiovascular irAEs, immunotherapy should be permanently discontinued. The experts have recommended a pulsed dose of methylprednisolone (1 g/day for 3–5 days) in severe or life-threatening situations (Thompson et al., 2021). As for the re-administration of PD-1/PD-L1 inhibitors, the society for immunotherapy of cancer (SITC) recommended that patients with grade 4 irAEs (moderate to severe decompensation, life-threatening conditions) should permanently discontinue the ICIs treatment. But SITC also suggested high-dose corticosteroids for severe conditions (Puzanov et al., 2017). The European Society for Medical Oncology (ESMO) recommended that when myocarditis was suspected, the patient should be admitted to the hospital and immediately start using high-dose prednisone (methylprednisolone 1,000 mg/day). If the condition worsened, consider adding another immunosuppressive drug (mycophenolate mofetil or tacrolimus) (Curigliano et al., 2020). In a multi-center registration report, Mahmood found that initial high-dose steroids were seemly more effective for patients, with a lower rate of MACE (Mahmood et al., 2018). Mycophenolate mofetil, tacrolimus, infliximab, and anti-thymocyte globulin have also been reported to be combined with steroids to alleviate the symptoms (Brahmer et al., 2018; Agrawal et al., 2019; Curigliano et al., 2020; Guo et al., 2020; Thompson et al., 2021).

## 7 Conclusion

PD-1/PD-L1 inhibitors associated with myocarditis are a rare and fatal complication. Given this, it is difficult to carry out high-quality large-scale prospective or retrospective clinical trials. Case reports or case series reports are effective and convenient ways for us to know the disease, but learning from the literature usually has poor timeliness. Patient follow-up information cannot be instantly tracked. Databases such as VigiBase allow us to know the incidence of immune-related myocarditis, but there is no information on the clinical manifestations, onset time, and therapy of the disease detailed mentioned in case reports, which are the keys to the diagnosis and treatment of myocarditis (Salem et al., 2018; Rubio-Infante et al., 2021). It is urgent to establish a public database of rare diseases, which records the primary disease, complications, onset time, treatment and survival status, etc., and can also real-time update the follow-up information, so that the public can dynamically evaluate the treatment of the disease.

Until now there are no specific clinical manifestations or laboratory biomarkers for PD-1/PD-L1 inhibitors associated myocarditis (Puzanov et al., 2017; Brahmer et al., 2018; Curigliano et al., 2020; Thompson et al., 2021). According to the location of myocarditis, the electrocardiogram may have nonspecific manifestations such as conduction block or ST-T changes. Echocardiography can accurately reflect the motion state of the ventricular wall, but in the early stage of the disease, when the left ventricular systolic function is normal, the manifestation that ultrasonography can provide us is unpredictive for myocarditis. CMR can find the structural abnormality of myocardial tissue and measure the ejection function of the heart. Compared with ultrasound, CMR has better accuracy and sensitivity. EMB is the gold standard for the diagnosis of immune-related myocarditis, which is characterized by a large number of immune T-cell infiltration. Some patients with deteriorated cardiac conditions cannot afford to suffer the EMB that may result in some fatal complications. Myocarditis is usually an acute course with severe symptoms. Early diagnosis and treatment are important measures to improve patient prognosis and reduce patient mortality. We proposed two strategies to help diagnose and treat PD-1/PD-L1 inhibitors associated myocarditis. First, an assessment of patients’ cardiovascular risk factors and history of cardiac adverse events should be completed before the administration of PD-1/PD-L1 inhibitors. Under this circumstance, we can get patients’ baseline index that can help evaluate the deteriorated extent of cardiac function. Second, for patients who cannot tolerate EMB, we can score the examinations or biomarkers related to the heart, such as electrocardiogram, echocardiogram, BNP, troponin, and CMP. When the score reaches a certain level, we can try to use steroids, with the hypothesis that the patients are likely to have myocarditis.

PD-1/PD-L1 inhibitors leading to T-lymphocyte infiltration is the histological foundation of immune myocarditis. It has been testified that PD-L1 can be expressed on myocardial tissue, so that PD-1/PD-L1 inhibitors may disturb the homeostasis that already existed. Considering the extremely low incidence of PD-1/PD-L1 inhibitors associated myocarditis, histological susceptibility may not be reasonable for the happening. Myocardial immune microenvironment changes, like, the unbalance of Tregs and Teffs, unnormal levels of cytokines, and macrophages polarization, will lead to T-lymphocyte infiltration, causing the fundamental pathogenesis of myocarditis. Francisco et al. have found PD-L1 can regulate the induction and function of Tregs by many signal pathways, like the down-regulation of phospho-Akt, mTOR, S6, and ERK2 and the upregulation of PTEN (Francisco et al., 2009). Zhang thought that PD-1 may play a key role in maintaining the balance and activation threshold of Tregs and Teffs, with FoxP3 maintaining the function of Tregs (Zhang et al., 2016). TNF-α, IFN-γ, and other cytokines can differently influence the expression of PD-1 or PD-L1 leading to the final phenotype of T-lymphocyte infiltration (Rodig et al., 2003; Seko et al., 2007; Zheng et al., 2018; Theivanthiran et al., 2020; Finke et al., 2021). Lv et al., have found that protein α-isoform of myosin heavy chain (α-MyHC, a kind of cardiac antigen) encoded by Myb6 let mice and humans receive the immune tolerance with the help of medullary thymic epithelial cells (mTECs) and peripheral lymphoid stromal cells, preventing from T-lymphocyte infiltration myocarditis (Lv et al., 2011). Tarrio has described that a αMyHC peptide can successfully induce the susceptibility of autoimmune myocarditis (with CD4^+^ and CD8^+^ T-lymphocyte infiltration) in PD-1 deficient mice (Tarrio et al., 2012). Tsuruoka et al. (2020) have found that the concurrent and subsequent administration of mPD1ab with MyHC-α fragment can manifest opposite effects on immune cell infiltration. Given that, whether thymic related auto-immune tolerance is the key point for immune myocarditis triggered by PD-1/PD-L1 inhibitors? Future consideration: 1) More studies should focus on the genetic level to investigate the genetic predisposition in myocarditis case; 2) exosome and cytokines are the hoping territories for the mechanism investigation because there are the messengers of information in different pathways; 3) immune microenvironment is the core for understanding and investigating the myocarditis. In addition, we have tried to summarize the potential mechanism of PD-1/PD-L1 inhibitors related in Table 4 from five different aspects based on the current published studies.

**TABLE 4:** Summary of potential or related mechanisms for PD-1/PD-L1 associated myocarditis.

Pathogenesis	Phenotype	Background	Authors
T-lymphocyte infiltration			
	αMyHC peptide causing T-lymphocyte lnfiltration	PD-1 deficient mice	Tarrio et al. (2012)
	T-lymphocyte infiltration	Coadministration of ipilimumab and nivolumab in monkeys	Ji et al. (2019)
	PD-1 deficiency increases CD8^+^ T-cell-mediated cardiac inflammation and damage	PD-1 deficient mice	Tarrio et al. (2012)
	PD-1 deficiency augments CD4^+^ dependent experimental autoimmune myocarditis	PD-1 deficient mice	Tarrio et al. (2012)
T-cell homeostasis			
	Regulatory T cells increased	Coadministration of ipilimumab and nivolumab in monkeys	Ji et al. (2019)
	PD-L1 synergizes with TGF β to promote induced T reg cell conversion	PD-L1/L2 genetic deficient mice	Francisco et al. (2009)
	PD-L1 induced CD4+Foxp3+ T reg cells suppress CD4^+^ T eff cells	PD-L1/L2 genetic deficient mice	Francisco et al. (2009)
	PD-L1 enhances and maintains Foxp3 expression on induced T reg cell and augments suppression at low T reg/T eff cell ratios	PD-L1/L2 genetic deficient mice	Francisco et al. (2009)
	PD-L1 deficiency leads to impaired T reg cell conversion	PD-L1/L2 genetic deficient mice	Francisco et al. (2009)
	PD-L1 antagonizes the Akt–mTOR signaling cascade during the induction of induced T reg cells	PD-L1/L2 genetic deficient mice	Francisco et al. (2009)
	IFN-γ from pathogenic CTLs increased expressed level of cardiac endothelial PD-L1	C57BL/6 CMy-mOva transgenic mice with different genetic alteration	Grabie et al. (2007)
	PD-1 deficiency changes intracardiac inflammatory cytokine production (secreting more IFNγ and granzyme B, and less IL-10)	PD-1 deficient mice	Tarrio et al. (2012)
Cytokines			
	IL4, IL6, IL10, IFN-γ, and TNF-α increased	Coadministration of ipilimumab and nivolumab in monkeys	Ji et al. (2019)
	IFN-γ promote the expression of PD-L1 on cardiac myocytes	C3H/He mice treated with anti-PD-1 monoclonal antibody	Seko et al. (2007)
	Induction of PD-L1 mRNA in cultured cardiac myocytes by IFN-γ	C3H/He mice treated with anti-PD-1 monoclonal antibody	Seko et al. (2007)
	Increased the expression of IFN-γ, FasL, and CD40L	C3H/He mice treated with anti-PD-1 monoclonal antibody	Seko et al. (2007)
	FGL2 promotes the levels of BNP, IL-6, IL-17, and IFN-γ in sera of autoimmune myocarditis rats	Rats that received lentivirus carrying FGL2	Zheng et al. (2018)
	FGL2 downregulates PD-1 but upregulates RORγt and Foxp3 mRNA and protein levels in autoimmune myocarditis rats	Rats that received lentivirus carrying FGL2	Zheng et al. (2018)
	The expression of IL-17A, IFNγ, and the transcription factor RORγt increased	PD-1 deficient mice	Tarrio et al. (2012)
Synergistic effect of PD-1/PD-L1 and CTLA4			
	Ctla4 and Pdcd1 functionally interact in a gene dosage-dependent manner in myocarditis	Ctla4^tm1All^ mice were bred to Pdcd1 knockout mice	Wei et al. (2021)
	CTLA4 and PD-1 block CD28-mediated activation to increase in metabolism	Huaman peripheral blood	Chemnitz et al. (2004)
Macrophage-mediated inflammatory response			
	Exosomes derived from PD-1 inhibitor–treated macrophages exerted a pro-senescent effect by modulating the miR-34a-5p/PNUTS signaling pathway	Male C57/Bl6 mice	Xia et al. (2020a)
	PD-1 inhibitor exerted its effect in promoting M1 polarization and cardiac injury by modulating the miR-34a/KLF4 signaling pathway and inducing myocardial inflammation	Male C57/Bl6 mice	Xia et al. (2020b)
Other			
	Auto-antibodies against cardiac myosin	Coadministration of ipilimumab and nivolumab in monkeys	
	PD-L1 expression upregulated in the heart during cytotoxic T-lymphocytes-mediated cardiac inflammation	C57BL/6 CMy-mOva transgenic mice with different genetic alteration	Grabie et al. (2007)
	PD-L1 Controls polymorphonuclear leukocytes recruitment to the heart and resistance to CTL-induced cardiac injury	C57BL/6 CMy-mOva transgenic mice with different genetic alteration	Grabie et al. (2007)
	Polymorphonuclear leukocytes depletion can reverse susceptibility to lethal myocarditis in the setting of PD-L1/2 deficiency	C57BL/6 CMy-mOva transgenic mice with different genetic alteration	Grabie et al. (2007)

For patients diagnosed with PD-1/PD-L1 inhibitors related, corticosteroid treatment is a recommended principle in the guidelines of the NCCN, SITC, ESMO, and American Society of Clinical Oncology. In the American Society of Clinical Oncology, NCCN, and ESMO guidelines, prompt steroids should be used initially. NCCN guideline-recommended high-dose steroids (1 g/day, IV) used for 3–5 days, while ESMO failed to detail the period of high-dose steroids usage, but suggested that steroids should be used until the symptoms improved and biomarker normalized. The American Society of Clinical Oncology recommended that the ways of administration of high-dose steroids (1–2 mg/kg) mainly depended on the severity of symptoms (Brahmer et al., 2018; Curigliano et al., 2020; Thompson et al., 2021). The consensus of SITC suggested that steroids treatment (2 mg/kg/day, IV) were used under the condition that patients with moderately abnormal testing or symptoms with mild activity (Puzanov et al., 2017). As for the PD-1/PD-L1 inhibitor rechallenge, those guidelines or consensuses have different opinions. NCCN and the American society of clinical oncology guidelines suggested the permanent discontinuation of PD-1/PD-L1 inhibitors in the setting of grade 2–4 (Brahmer et al., 2018; Thompson et al., 2021). While the consensus of SITC considered that it was reasonable to stop immune checkpoint inhibitors forever for patients in grade 4 (Puzanov et al., 2017). In the ESMO guideline, the PD-1/PD-L1 inhibitor rechallenge should balance the benefits and risk for patients, and if necessary very close surveillance of anti-PD-1 agents should be carried out (Curigliano et al., 2020). Given the diagnostic difficulty of PD-1/PD-L1 inhibitor-associated myocarditis, patients with suspected heart failure or severe cardiac symptoms are highly recommended to receive steroids, because the EMB may be a dangerous intervention in that setting. And in some situations, doctors will make the diagnosis of PD-1/PD-L1 associated myocarditis, according to the cardiac imaging performance, clinical symptoms and the usage of PD-1/PD-L1 inhibitors (Gibson et al., 2016; Mehta et al., 2016; Semper et al., 2016; Frigeri et al., 2018; Monge et al., 2018; Thibault et al., 2018; Agrawal et al., 2019; Charles et al., 2019; Esfahani et al., 2019; Khoury et al., 2019; Salem et al., 2019). Steroid therapy is not only a treatment method but also a diagnostic method for myocarditis if the patients’ symptoms improved. If there are no obvious improvements in clinical symptoms under steroid usage, immunosuppressive agents should be used as second-line treatment. There are two dilemmas we have to find the answers to. First, the anti-tumor efficacy of PD-1/PD-L1 inhibitors will be compromised by the usage of steroids or immunosuppressive agents? Second, when and how to judge the rechallenge under the setting that PD-1/PD-L1 inhibitors achieve promising anti-tumor effect? For the first question, we think that it is wise to use steroid or immunosuppressive agents to treat myocarditis under the characteristics that are acute and more lethal. Unfortunately, there is no consensus on the second question.

In this article, we reviewed the epidemiological manifestations, clinical characteristics, diagnosis, pathogenesis, and treatment of PD-1/PD-L1 inhibitor-related myocarditis and summarized the reported myocarditis cases in order to outline the clinical symptoms. Immuno-myocarditis is a rare but severe adverse event of PD-1/PD-L1 inhibitirs related treatment, usually with an early onset. Biomarkers, like BNP or troponin, ECG, and MCR can help diagnose immuno-myocarditis. Exploring the possible mechanisms of pathogenesis from the histological level, cellular level, and molecular transcription level found that T-lymphocyte infiltration was the basis of myocarditis phenotype. Macrophages mediated immune-response; synergistic effect of PD-1/CTLA4 may be the catalysts in this process. The core treatment of PD-1/PD-L1 inhibitor-related myocarditis is to suppress the over-activated T lymphocytes and corticosteroids are the first-line immunosuppressant.
